# The association of body composition and fat distribution with hypertension in community-dwelling older adults: the Bushehr Elderly Health (BEH) program

**DOI:** 10.1186/s12889-023-16950-8

**Published:** 2023-10-13

**Authors:** Mohammad Mehdi Khaleghi, Ali Jamshidi, Sima Afrashteh, Hadi Emamat, Akram Farhadi, Iraj Nabipour, Zahrasadat Jalaliyan, Hasan Malekizadeh, Bagher Larijani

**Affiliations:** 1 Student Research Committee, Bushehr University of Medical Sciences, Bushehr, Iran; 2 The Persian Gulf Tropical Medicine Research Center, The Persian Gulf Biomedical Sciences Research Institute, Bushehr University of Medical Sciences, Bushehr, Iran; 3 Department of Nutritional Sciences, Faculty of Health and Nutrition, Bushehr University of Medical Sciences, Bushehr, Iran; 4 Department of Biostatistics and Epidemiology, Faculty of Health and Nutrition, Bushehr University of Medical Sciences, Bushehr, Iran; 5 The Persian Gulf Marine Biotechnology Research Center, The Persian Gulf Biomedical Sciences Research Institute, Bushehr University of Medical Sciences, Bushehr, Iran; 6 School of Medicine, Bushehr University of Medical Sciences, Bushehr, Iran; 7 Endocrinology and Metabolism Research Center, Endocrinology and Metabolism Clinical Sciences Institute, Tehran University of Medical Sciences, Tehran, Iran

**Keywords:** Hypertension, Adiposity, Android obesity, Gynoid obesity, Elderly, Central obesity

## Abstract

**Background:**

A significant proportion of the global burden of disability and premature mortality has caused by hypertension. It seems that the relationship between obesity and hypertension is not only associated with excessive body fat mass (FM) but also with body adipose distribution patterns. The present study investigated the association between regional fat distribution using dual-energy X-ray absorptiometry and hypertension in older adults.

**Methods:**

This cross-sectional study was performed using the data from Bushehr Elderly Health Program (BEH) on a total of 2419 participants aged 60 and over. Hypertension was defined as SBP of at least 140 mmHg and/or DBP of at least 90 mmHg. SBP between 120 and 139 mmHg and/or a DBP between 80 and 89 mmHg were considered prehypertension. Participants underwent body composition measurement by dual-energy x-ray absorptiometry to analyze FM, fat-free mass (FFM) in trunk and extremities composition.

**Results:**

The results showed that 460 (19.02%) of participants had prehypertension, and 1,818 (75.15% ) had hypertension. The odds of having prehypertension (OR: 1.06, 95%CI: 1.01–1.12) and hypertension (OR: 1.08, 95%CI: 1.03–1.13) increased with a rise in total body FM percentage. Moreover, people with a higher FM to FFM ratio had increased odds of being prehypertensive (OR: 9.93, 95%CI: 1.28–76.99) and hypertensive (OR: 16.15, 95%CI: 2.47-105.52). Having a higher android to gynoid FM ratio was related to increased odds of being prehypertensive and hypertensive.

**Conclusions:**

This study showed that a higher body FM, particularly in the android region, is associated with higher odds of having hypertension in older adults.

## Background

Non-communicable diseases (NCDs) are the notable cause of mortality, increasing over the recent two decades. Four major NCDs, including cancers, diabetes, chronic respiratory diseases, and cardiovascular diseases (CVDs), were responsible for 70% of the 56.4 million deaths worldwide in 2015, according to World Health Organization (WHO) [1]. A significant proportion of the global burden of cardiovascular disease, disability, and premature mortality has caused by hypertension. More than one billion people suffer from hypertension, of which 75% live in low- and middle-income countries [2]. In 2019, high systolic blood pressure was accountable for about 30.6% of mortalities in Iran [3].

Although high blood pressure can be controlled by many demonstrated and effectual pharmacological treatments and lifestyle modifications (such as salt intake, smoking, alcoholic drinks, sleep duration, obesity, and abdominal obesity), it remains highly prevalent [4]. Adipose tissue is an endocrine organ and active tissue for energy homeostasis which synthesizes and secrets hormones such as leptin and adiponectin and inflammatory mediators like TNF-alpha and Interluekin-6. Adipose tissue dysfunction can be caused by obesity, which is recognized by dysregulation in secreting many vasoactive adipokines and anti-inflammatory cytokines, changing secretory profiles, mitochondrial dysfunction, and tissue inflammation [5, 6]. It seems that the relationship between obesity and hypertension is not only associated with excessive body fat but also with body adipose distribution patterns [7].

Although body mass index (BMI) is the most typical obesity indicator to predict the risk of CVDs, it suffers from the inability to determine tissue type (muscle or adipose tissue) or regions of body mass distribution [8, 9]. Since body composition assessment plays a crucial role in studying human metabolism and physiology, it is preferable to use segmental measurements that can indicate the effect of obesity more accurately [10]. A study by Yano et al. showed that excessive fat accumulation in the visceral area, but not subcutaneous adipose tissue, was related to higher mean blood pressure in the short- and long-term and decreased long-term blood pressure variability [11]. In addition, another study demonstrated that trunk fat mass was the predominant contributor to a higher SBP and DBP, while leg fat mass had the opposite relationship with BP [12]. Dual-energy X-ray absorptiometry (DXA) scan is a gold standard that is able to differentiate between fat tissues and measure body fat distribution accurately and objectively [13, 14].

There is a lack of understanding regarding the precise relationship between regional fat distribution, prehypertension, and hypertension risk in older adults. This gap is especially significant considering that conventional measures such as body mass index (BMI) do not provide sufficient precision for this specific age group. To address this, the study aims to investigate the relationship between regional fat distribution (using DXA), and hypertension in the older adult population of southern Iran based on the Bushehr Elderly Health (BEH) program. By examining specific patterns of fat accumulation, the research aims to provide insights for targeted interventions and preventive strategies.

## Methods

### Design study and participants

**Fig. 1:**
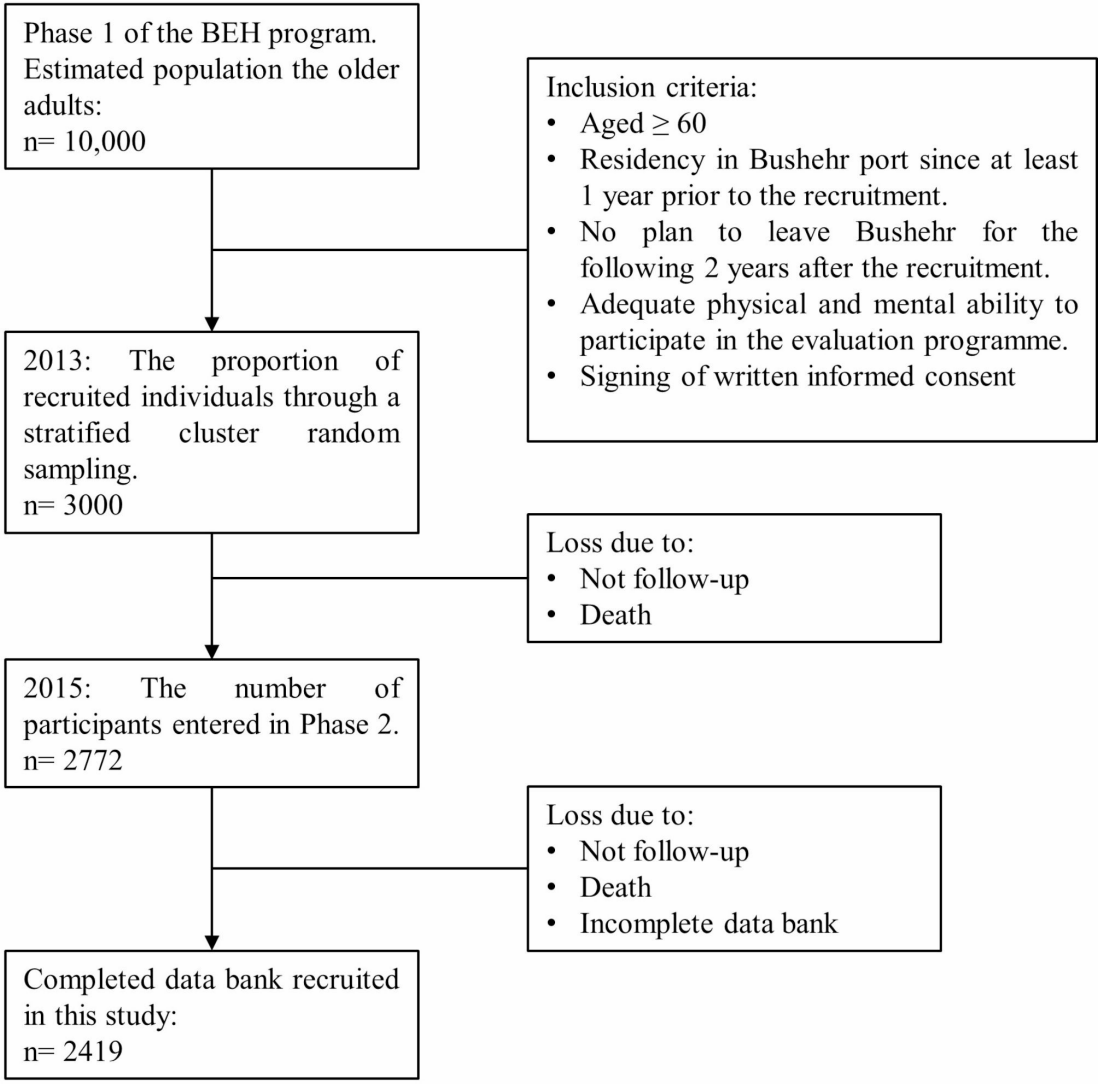
Flow chart of enrolment in the Bushehr Elderly Health (BEH) program

This cross-sectional study was performed using the data from Bushehr Elderly Health Program (BEH), a prospective demographic cohort study conducted in Bushehr, Southern Iran. Detailed protocols have been reported previously, including the study design and methodology for the BEH program [15, 16]. In summary, the first phase of the BEH program commenced in March 2013 to investigate the prevalence of cardiovascular risk factors and their relationship with adverse cardiovascular events in the older adult population. A total of 3000 participants aged 60 and over were recruited by stratified random sampling method. Subjects were selected if they lived in Bushehr for at least one year before the recruitment with no plan to leave in the following two years, had adequate physical and mental ability to cooperate in the study, and signed written consent. The second stage of the first phase of the study was completed in 2015–2018. The participation rate in the second phase was 81%. Finally, all the 2419 individuals with completed databanks were included in this study (Fig. 1).

The database included demographic status, general health, mental and functional health, lifestyle, and history of drug use, which was collected using valid forms and questionnaires. The Ethics Research Committee of Bushehr University of medical sciences (IR.BPUMS.REC.1401.173) and the Endocrinology and Metabolism Research Institute of Tehran University of Medical Sciences approved the study protocol.

### Blood pressure

As our primary outcome, systolic blood pressure (SBP) and diastolic blood pressure (DBP) were measured twice using a standard mercury sphygmomanometer in the right arm after 15 min of rest in a sitting position with a time interval of 10 min. The average of the two measurements was taken as the participant’s blood pressure. Individuals were classified according to SBP and DBP levels into normal blood pressure, prehypertension, and hypertension. Hypertension was defined as SBP of at least 140 mmHg and/or DBP of at least 90 mmHg or taking antihypertensive medications. SBP between 120 and 139 mmHg and/or a DBP between 80 and 89 mmHg were considered prehypertension [7].

### Laboratory evaluations

After 8–10 h of fasting, 25 ml of venous blood samples were collected by a trained nurse and stored in coded containers for further testing. Laboratory tests included fasting blood sugar (FBS) (mmol/L) and blood lipid profile (total cholesterol, HDL-c, LDL-c, and triglycerides) (mmol/L). All the processes were executed by professional personnel and advanced tools. Laboratory FBS and blood lipid profile testing were measured by enzymatic colorimetric method using a Pars Azmun kit (Pars Azmun, Karaj, Iran).

### Anthropometric and clinical assessments

Anthropometric indexes were quantified under the standard protocol by wearing light clothing and removing shoes. A fixed stadiometer and a digital scale were used to measure height and weight. A flexible, constant tension tape was used to determine neck, waist and hip circumferences at the level of the umbilicus and the widest part over the buttocks, respectively. Body mass index (BMI) was calculated by dividing the weight (kg) by height squared (m^2^). Participants underwent body composition measurement by dual-energy x-ray absorptiometry (DXA, Discovery WI, Hologic Bedford, Virginia, USA) to analyze fat mass, muscle mass, and head, trunk, and extremities composition.

### Android and gynoid regions

The Android region height was considered equal to 20% of the distance from the pelvis horizontal line to the neckline. The lower boundary of the Android region coincided with the pelvic horizontal line, and the lateral boundaries of the Android region coincided with the arm lines. The Gynoid region height was considered equal to twice the height of the Android region. The upper limit of the Gynoid region was below the pelvic horizontal line by 1.5 times the Android region’s height, and the Gynoid region’s lateral boundaries coincided with the arm lines [17].

### Other covariates

Socio-demographic information, such as age, gender, marital status, physical activity, income levels, education, occupation, smoking history, medical history, and drug use, were collected through interviews. The average physical activity level, encompassing sports, work, and leisure time, was determined by assessing the metabolic equivalent of tasks over a 24-hour period using the International Physical Activity Questionnaire (IPAQ). The widely adopted self-report tool is designed to measure the physical activity levels of individuals and has been validated within the Iranian population [15, 18, 19]. The level of physical activity was categorized into three classes: no activity and sedentary: ≤1.39; low active: 1.4–1.59; active: ≥1.6 (MET/24 h) [20, 21].

### Data analysis

Categorical variables of general characteristics were presented as frequencies and percentages, whereas continuous variables were expressed as mean values and standard deviation (SD). Differences between the groups were evaluated using a one-way ANOVA for continuous and Chi-square for categorical variables. The odds ratio and 95% confidence interval were calculated through multinomial logistic regression analyses to determine the association of prehypertension and hypertension with anthropometric and body composition indices.

Relevant confounders were selected based on an extensive literature search. Firstly, their clinical and pathophysiological association with the desired outcome and exposures was assessed using univariate regression models. Then, statistically significant covariates, which have clinical implications, were included in the multivariable logistic regression models. Analytical models were set as model **crude**; **model 1**: adjusted for age; **model 2**: adjusted for age and gender; **model 3**: adjusted for age, gender, marital, education, job, physical activity, smoking, income, kidney stone, diabetes, BMI, TG, TC, LDL, HDL. All analyses were done in Stata MP (version 17). P-value < 0.05 was taken as statistically significant for all analyses.

## Results

The general characteristics of the participants are shown in Table 1. This study included 2,419 participants, 460 (19.02%) of whom had prehypertension, and 1,818 (75.15%) had hypertension. The mean age in people with hypertension was significantly higher (69·65 ± 6·53 years vs. 68·45 ± 5·58 years) than the mean age in the group with normal blood pressure. While the prevalence of prehypertension was higher in men than in women (21.6% vs. 16.6%), the proportion of women with hypertension was higher than in men (76.7% vs. 73.5%). 68.7% of people with hypertension had a history of smoking (50.6% past smokers, 18.1% current smokers). The prevalence of hypertension was lower in those still employed than in those who were retired or unemployed (66.6% vs. 74.4% and 76.7%, respectively). Also, being physically sedentary or not active and having diabetes was related to a higher prevalence of hypertension. No statistical differences were observed in the prevalence of prehypertension and hypertension regarding having diseases such as stroke, chronic renal failure, thyroid diseases, or lung disease. There was also a group of individuals that they were not consuming any antihypertensive medicine although 22% and 71% of them were suffering from prehypertension and hypertension, respectively, as untreated individuals (Table 1).

**Table 1:** Characteristics of the study participants according to blood pressure groups. (n = 2419)

Characteristics	Categories	Normal 141 (5.83%)	Prehypertension 460 (19.02%)	Hypertension 1,818 (75.15%)	*P-value* ^*^
		*No. (%)*	*No. (%)*	*No. (%)*	
Age (year)		68.45 ± 6.82	68.45 ± 5.58	69.65 ± 6.53	< 0.001
Gender	Male	57 (4.90)	251 (21.60)	855 (73.50)	0.003
	Female	84 (6.70)	209 (16.60)	963 (76.70)	
Education	Illiterate	43 (5.38)	129 (16.15)	627 (78.47)	0.063
	Under diploma	62 (5.64)	220 (20.02)	817 (74.34)	
	Diploma & academic	36 (6.91)	111 (21.31)	374 (71.79)	
Marital status	Married	100 (5.39)	370 (19.92)	1,387 (74.69)	0.041
	Others (Single, Divorced, Widowed)	41 (7.30)	90 (16.01)	431 (76.69)	
Occupation	Employed	8 (5.93)	37 (27.41)	90 (66.67)	0.023
	Retired	57 (5.30)	218 (20.28)	800 (74.42)	
	Unemployed	76 (6.29)	205 (16.96)	928 (76.76)	
Income level	Low	37 (26.24)	95 (20.65)	381 (20.96)	0.573
	Moderate	75 (53.19)	254 (55.22)	1,029 (56.60)	
	High	29 (20.57)	111 (24.13)	408 (22.44)	
Physical activity	No active &Sedentary	106 (5.68)	343 (18.39)	1,416 (75.92)	0.026
	Low active	29 (7.30)	73 (18.39)	295 (74.31)	
	Active	6 (3.82)	44 (28.03)	107 (68.15)	
Smoking	No	38 (26.95)	127 (27.61)	568 (31.24)	< 0.001
	Past smoking	51 (36.17)	211 (45.87)	921 (50.66)	
	Current smoking	52 (36.88)	122 (26.52)	329 (18.10)	
Stroke	Yes	9(11.10)	14(17.30)	58(71.60)	0.117
	No	132(5.60)	446(19.10)	1760(75.30)	
Diabetes	Yes	24 (3.36)	107 (14.99)	583 (81.65)	< 0.001
	No	117 (6.86)	353 (20.70)	1,235 (72.43)	
kidney stone	Yes	6 (2.83)	40 (18.87)	166 (78.30)	0.142
	No	135 (6.12)	420 (19.05)	1,650 (74.83)	
Chronic renal failure	Yes	1(2.90)	4(11.80)	29(85.3)	0.387
	No	139(5.80)	455(19.10)	1789(75.10)	
Thyroid disease	Yes	12(5.20)	33(14.30)	185(80.40)	0.134
	No	129(5.90)	427(19.50)	1633(74.60)	
Lung disease	Yes	6(6.10)	18(18.20)	75(75.80)	0.974
	No	135(5.80)	442(19.10)	1743(75.10)	
Antihypertensive medication	Yes	0(0.00)	1(0.10)	1281(99.90)	< 0.001
	No	141(12.40)	459(40.40)	537(47.20)	
Lipid-lowering medication	Yes	31(4.00)	97(12.60)	641(83.40)	< 0.001
	No	110(6.70)	363(22.00)	1177(71.30)	
Diabetes medication	Yes	24(3.40)	105(14.70)	584(81.90)	< 0.001
	No	117(6.90)	355(20.80)	1234(72.30)	
**Anthropometric measurement**					
BMI (kg/m2)		25.08 ± 5.05	26.33 ± 4.19	28.00 ± 4.93	< 0.001
NC (cm)		35.01 ± 3.60	36.67 ± 3.52	37.23 ± 3.60	< 0.001
WC (cm)		92.03 ± 12.92	95.98 ± 11.05	99.91 ± 11.85	< 0.001
HC (cm)		98.65 ± 9.95	100.80 ± 8.38	103.30 ± 10.44	< 0.001
WHR		0.93 ± 0.07	0.95 ± 0.07	0.96 ± 0.09	< 0.001
**Dual-energy X-ray absorptiometry**					
LBM (kg)		38.60 ± 8.07	41.66 ± 8.69	41.08 ± 10.35	0.005
ASM (kg)		14.79 ± 3.27	16.03 ± 3.64	15.91 ± 3.64	0.001
Total fat mass (kg)		22.03 ± 8.57	23.84 ± 7.46	26.42 ± 7.92	< 0.001
Total fat percentage (%)		35.24 ± 9.32	35.90 ± 8.24	38.21 ± 7.86	< 0.001
FM to LBM ratio		0.57 ± 0.22	0.64 ± 0.21	0.58 ± 0.20	< 0.001
**Laboratory**					
TC, mmol/L		184.96 ± 47.36	186.93 ± 44.16	180.84 ± 43.87	0.022
TG, mmol/L		118.10 ± 62.92	131.48 ± 77.14	138.50 ± 69.03	0.001
HDL-c, mmol/L		47.16 ± 10.77	46.59 ± 11.90	45.68 ± 11.07	0.123
LDL-c, mmol/L		114.54 ± 42.17	113.99 ± 36.36	107.93 ± 37.60	0.002
FBS, mmol/L		99.65 ± 41.69	103.84 ± 43.61	107.38 ± 42.37	0.046

• Data are reported as mean and SD for continuous variables, and as proportion and percentage for categorical variables

P-values were derived from the Chi-Squared test for categorical variables and One-way ANOVA for continuous variables represents differences between groups

• * The significance level was considered P < 0.05.•

Abbreviation note: **BEH**: Bushehr elderly health; **BMI**: body mass index; **NC**: neck circumferences; **WC**: Waist circumferences; **HC**: hip circumferences; **WHR**: Waist to hip ratio; **FM**: fat mass; **FFM**: fat-free mass; **LBM**: lean body mass, **ASM**: appendicular skeletal muscle, **TC**: total cholesterol; **TG**: triglyceride; **HDL-c**: high-density lipoprotein cholesterol; **LDL-c**: low-density lipoprotein cholesterol; **FBG**: fasting blood glucose.

According to Table 1, normotensive people had significantly lower mean weight, BMI, NC, WC, HC, and WHR in comparison with prehypertensive and hypertensive groups. In addition, DXA analysis showed that mean Lean Body Mass (LBM), Appendicular Skeletal Muscle (ASM), total fat percentage, and FM to LBM ratio were lower in the group with normal blood pressure.

In the examination of the laboratory data, it was seen that while TC, HDL, and LDL were statistically lower in participants with hypertension, TG and FBS were higher (Table 1).

According to Table 2, the hypertension group had the highest mean Fat Mass Percentage (FMP) and Fat Mass (FM) to Fat-Free Mass (FFM) ratio in the limbs and trunk regions and the total body. Also, the trunk-to-limbs FM ratio and Android-to-Gynoid FM ratio in the HTN group were higher than the normal and pre-HTN group (p < 0.001).

**Table 2:** The comparison of mean adiposity index according to blood pressure groups

		Total (n = 2,419)	Normal (n = 141)	Prehypertension(n = 460)	Hypertension (n = 1,818)	p-value
Limb	FMP	38.18 ± 9.77	36.27 ± 10.64	36.45 ± 9.84	38.77 ± 9.61	< 0.001
	FM to FFM ratio	0.66 ± 0.27	0.61 ± 0.26	0.61 ± 0.26	0.67 ± 0.27	< 0.001
Trunk	FMP	38.94 ± 8.05	36.02 ± 9.76	37.14 ± 8.43	39.61 ± 7.67	< 0.001
	FM to FFM ratio	0.66 ± 0.21	0.59 ± 0.23	0.61 ± 0.21	0.68 ± 0.21	< 0.001
Total	FMP	37.59 ± 8.01	35.26 ± 9.29	35.91 ± 8.22	38.19 ± 7.76	< 0.001
	FM to FFM ratio	0.62 ± 0.21	0.57 ± 0.22	0.58 ± 0.20	0.64 ± 0.20	< 0.001
Trunk to limb FM ratio		1.35 ± 0.27	1.27 ± 0.23	1.33 ± 0.26	1.36 ± 0.27	< 0.001
Android to Gynoid FM ratio		1.12 ± 0.17	1.03 ± 0.18	1.11 ± 0.17	1.13 ± 0.17	< 0.001

Values are reported as mean and SD.

P-values derived were derived from analysis of one-way ANOVA.

The significance level was considered P < 0.05.

Abbreviation note: **FMP**: Fat Mass Percentage; **FM**: fat Mass; **FFM**: Fat-Free Mass

Table 3 represents the relationship of adiposity indexes with prehypertension and hypertension among the older adults in this study. The odds of having prehypertension (OR: 1.06, 95%CI: 1.01–1.12; p = 0.009) and hypertension (OR: 1.08, 95%CI: 1.03–1.13; p = 0.001) increased with a rise in total body FM percentage in comparison with the normal group. Moreover, people with a higher FM to FFM ratio had increased odds of being prehypertensive (OR: 9.93, 95%CI:1.28–76.99; p = 0.028) and hypertensive (OR: 16.15, 95%CI: 2.47-105.52; p = 0.004). With a rise in the FM to FFM ratio in limbs, the risk of being prehypertensive (OR: 6.74, 95%CI: 1.39–32.73; p = 0.018) and hypertensive (OR: 7.69, 95%CI: 1.80-32.84; p = 0.006) increased; however, in the trunk area, the FM to FFM ratio was only associated with higher odds of hypertension (OR: 7.73, 95%CI: 1.60-37.32; p = 0.011).

**Table 3:** Adjusted association of adiposity index with prehypertension and hypertension in the Bushehr Elderly Health (BEH) program

			Normal	Prehypertension n = 460		Hypertension n = 1,818	
				OR,95%CI	P-value	OR,95%CI	P-value
Limb	FM (%)	Crude	Ref	1.00(0.98–1.02)	0.847	1.02(1.00-1.04)	0.003
		Model 1	Ref	1.00(0.98–1.02)	0.849	1.02(1.00-1.04)	0.003
		Model 2	Ref	1.06(1.03–1.09)	< 0.001	1.10(1.07–1.13)	< 0.001
		**Model 3**	**Ref**	**1.06(1.01–1.10)**	**0.005**	**1.06(1.02–1.10)**	**0.002**
	FM to FFM ratio	Crude	Ref	1.01(0.49–2.10)	0.962	2.44(1.26–4.73)	0.008
		Model 1	Ref	1.01(0.49–2.09)	0.965	2.54(1.31–4.90)	0.005
		Model 2	Ref	8.38(2.60-26.93)	< 0.001	28.69(9.92–82.96)	< 0.001
		**Model 3**	**Ref**	**6.74(1.39–32.73)**	**0.018**	**7.69(1.80-32.84)**	**0.006**
Trunk	FM (%)	Crude	Ref	1.01(0.99–1.03)	0.166	1.05(1.03–1.07)	< 0.001
		Model 1	Ref	1.01(0.99–1.03)	0.167	1.05(1.03–1.08)	< 0.001
		Model 2	Ref	1.06(1.03–1.09)	< 0.001	1.11(1.08–1.14)	< 0.001
		**Model 3**	**Ref**	**1.04(1.03–1.09)**	**0.032**	**1.06(1.02–1.10)**	**0.002**
	FM to FFM ratio	Crude	Ref	1.61(0.65–3.99)	0.301	6.46(2.82–14.82)	< 0.001
		Model 1	Ref	1.61(0.64–4.02)	0.302	7.37(3.20-17.01)	< 0.001
		Model 2	Ref	9.68(2.97–31.50)	< 0.001	66.16(22.32-196.09)	< 0.001
		**Model 3**	**Ref**	**4.50(0.81–24.91)**	**0.080**	**7.73(1.60-37.32)**	**0.011**
Total	FM (%)	Crude	Ref	1.00(0.98–1.03)	0.403	1.04(1.02–1.06)	< 0.001
		Model 1	Ref	1.00(0.98–1.03)	0.407	1.04(1.02–1.07)	< 0.001
		Model 2	Ref	1.07(1.04–1.11)	< 0.001	1.13(1.10–1.16)	< 0.001
		**Model 3**	**Ref**	**1.06(1.01–1.12)**	**0.009**	**1.08(1.03–1.13)**	**0.001**
	FM to FFM ratio	Crude	Ref	1.30(0.51–3.33)	0.575	4.98(2.12–11.72)	< 0.001
		Model 1	Ref	1.30(0.51–3.32)	0.580	5.46(2.32–12.84)	< 0.001
		Model 2	Ref	15.49(3.91–61.33)	< 0.001	117.18(33.19-413.63)	< 0.001
		**Model 3**	**Ref**	**9.93(1.28–76.99)**	**0.028**	**16.15(2.47-105.52)**	**0.004**
Trunk-to-limb FM ratio		Crude	Ref	2.20(1.05–4.63)	0.036	3.80(1.92–7.50)	< 0.001
		Model 1	Ref	2.21(1.05–4.66)	0.036	4.03(2.03–7.99)	< 0.001
		Model 2	Ref	1.57(0.69–3.54)	0.275	4.13(1.96–8.69)	< 0.001
		**Model 3**	**Ref**	**0.92(0.39–2.16)**	**0.861**	**1.78(0.81–3.89)**	**0.147**
Android-to-Gynoid FM ratio		Crude	Ref	11.80(3.96–35.11)	< 0.001	23.45(8.63–63.72)	< 0.001
		Model 1	Ref	12.69(4.17–38.55)	< 0.001	29.01(10.45–80.54)	< 0.001
		Model 2	Ref	8.62(2.70-27.44)	< 0.001	39.48(13.53-115.13)	< 0.001
		**Model 3**	**Ref**	**2.62(0.68–10.13)**	**0.160**	**5.12(1.45–18.08)**	**0.011**

Data are presented as odds ratios (ORs) and 95% confidence intervals (95% CIs) derived from a multinomial logistic regression model.

**The final models are bolded.**

The significance level was considered P < 0.05.

**Models**:

Crude

Model 1 adjusted for age.

Model 2 adjusted for age + gender.

Model 3 adjusted for age, gender, marital status, education, job, physical activity, smoking, income, kidney stone, diabetes, lung disease, Stroke, Thyroid disease, Chronic Renal Failure and Cardiometabolic factors (BMI, TG, TC, LDL, and HDL).

Abbreviation note: **BEH**: Bushehr elderly health; **BMI**: body mass index; **FM**: fat mass; **FFM**: fat-free mass, **TC**: total cholesterol; **TG**: triglyceride; **HDL-c**: high-density lipoprotein cholesterol; **LDL-c**: low-density lipoprotein cholesterol; **FBG**: fasting blood glucose.

Regarding the relationship of fat distribution, having a higher android to gynoid FM ratio was related to increased odds of being prehypertensive and hypertensive in the crude model. In addition, the crude model’s FM ratio of the trunk to the limbs had a positive relationship with a higher odds of having prehypertension and hypertension. However, after considering confounders in the full model, the change was only significant for the association of Android-to-gynoid FM ratio and having hypertension (OR: 5.12, 95%CI: 1.45–18.08; p = 0.011).

## Discussion

Hypertension has been one of the significant public health issues in the last century, especially in older adults. The current study aimed to evaluate the association between body composition, emphasizing body fat distribution and hypertension in older adults. The result showed that body fat in the whole body and the regions of the trunk and limbs is closely related to being prehypertensive and hypertensive. In addition, the older adults with a higher Android to Gynoid fat mass ratio had an increased odds of developing hypertension in the Bushehr Elderly Health (BEH) program study.

The mean of age in the group with hypertension was higher than the group of normal blood pressure. Our results showed that 68.7% of hypertensive people were currently using smoke or used to consume it in the past. The previous studies indicated that life-course-adjusted smoking consumption was a major contributor to the increased risk of incidence of hypertension [22]. In addition, a sedentary lifestyle was also more prevalent in hypertensive individuals in our study. As a modifiable factor, physical activity has been shown to be a related intervention to prevent hypertension. It has been reported that people with active work styles tend to have lower blood pressure [23].

Our descriptive data indicated that individuals with higher education or employment appeared to have a lower prevalence of hypertension compared to those with no education or who were unemployed. However, it is important to note that the observed differences do not reach statistical significance for education levels. Although education’s impact on hypertension is reported to be limited in high-income countries, a meta-analysis has revealed that lower educational levels are associated with higher hypertension prevalence/incidence [24]. Being unemployed has also been identified as a factor for a 1.65-fold increase in the risk of hypertension [25].

### Fat mass percentage and fat mass to free-fat mass ratio

In this study, older adults with higher fat mass percentage and fat mass to fat-free mass ratio were more susceptible to prehypertension and hypertension. Our results showed that with each percent increase in fat mass percentage in the whole body, the odds of having prehypertension and hypertension increase by 6% and 8%, respectively. Moreover, the fat to fat-free mass ratio in the whole body was related to a 9.9-fold and 16.1-fold higher odds of having prehypertension and hypertension per unit increase, respectively. The association trend for regional fat distribution (namely limbs and trunk) and hypertension also was the same as the whole body.

Consistent with our results, Previous studies have stated that higher body fat was related to the increased prevalence of cardiometabolic diseases [26–28]. In addition, the odds of being metabolically abnormal were nearly tripled in people with elevated body fat compared to people with lower body fat [26]. Takase et al. also reported that a higher fat mass index was related to the increased prevalence of hypertension, which could be because of increased insulin resistance, raised sympathetic tone, and renin-angiotensin-aldosterone system abnormalities [29]. The study of Saito et al. assessed the higher body fat percentage variability related to a higher incidence of hypertension. They reported that hypertrophy of adipocytes is associated with inflammatory cytokines dysregulation, which could indicate the potential mechanism of body fat percentage fluctuation effect on cardiovascular risk [30]. Also, a lower basal metabolic rate can be related to a lower muscle mass. Muscle loss can enhance inflammation and oxidative pathways, leading to diabetes, material stiffness, and hypertension. Previous studies reported a higher lean body mass in the upper limbs and torso is a protective factor against metabolic diseases [10] and sarcopenic obesity, characterized by the presence of low muscle mass and strength combined with high levels of adiposity, is linked to elevated cardiovascular risk factors and a higher mortality risk among older adults [31, 32]. However, a study by Ye et al. showed an opposite results. They stated that after accounting for various potential confounding factors such as body fat mass and fat distribution, arm lean body mass (LBM) could emerge as a risk factor for increased blood pressure (BP) and hypertension [33]. Moreover, the relationship between various adiposity indexes (such as body mass index, waist circumference, and waist-to-height ratio) with hypertension has been revealed, and central adiposity indicators seem to be more associated with the risk of hypertension [34].

### Android to gynoid fat mass ratio

In this study, the fat mass ratio in the area of android to gynoid was positively associated with a rise in the prevalence of hypertension in older adults. We observed a 5.1-fold increase in the odds of hypertension per unit increase in the android-to-gynoid fat mass ratio in the fully adjusted model. Our research results are consistent with those previously reported in the existing literature [33, 35].

DXA analysis is a superior method to accurately determine body fat’s amount and distribution to the traditional methods such as skinfold or waist circumference [17, 36]. Gynoid refers to a relative excess of fat in the hips and thighs, while the android type illustrates excess upper-body fat [37]. The association of the android-to-gynoid ratio with metabolic and cardiovascular diseases has been reported, which can be explained by the crucial role of insulin in metabolism. The underlying linkage between diabetes, cardiovascular diseases, and obesity can be insulin resistance [17]. Accumulated fat in upper-body regions (such as visceral and epicardial adipose tissue) is a source of various proinflammatory cytokines like TNF-a and Interleukin-6. Conversely, higher gluteofemoral adipose tissue (GAT) mass plays a protective role in metabolic and cardiovascular diseases. GAT is related to lower cholesterol, glucose, and insulin levels, decreased vascular calcification, and arterial stiffness [5]. Yan et al. found that the accumulated trunk fat and decreased leg fat can be associated with increased blood pressure [36]. In addition, the study by Toss et al. showed peripheral obesity was associated with a higher level of adiponectin and lower insulin resistance in comparison with central obesity [38].

### The potential mechanisms linking adiposity and metabolic health

Adipose tissue is considered a metabolically active organ that constitutes 20% of the weight of a normal healthy person. Since adipose tissue plays a part in homeostasis, its prolonged disturbance can cause severe health issues [39]. The expanding ability of adipose tissue is one of the major factors in causing metabolic dysregulation. Adipose tissue has limited hyperplasia, so by reaching their limitation, the cells will hypertrophy. This process, which is regulated by genetics and environmental factors, leads to the excessive release of free fatty acids in blood circulation, followed by low-grade inflammation, atherogenesis, cardiovascular diseases, hypertension, insulin resistance, and dyslipidemia [5, 39]. Synthesizing and releasing diverse cytokines and hormones, including tumor necrosis factor-a, interleukin-6, C-reactive protein, leptin, adiponectin, and non-esterified fatty acids, is one of the significant factions of adipose organ. Low-grade inflammation, endothelial dysfunction, metabolic dysregulation, and free fatty acid circulation due to the excessive accumulation of fat mass can contribute to causing hypertension [2].

This study has some strengths and limitations. The present investigation has received significant advantages from a relatively substantial sample size obtained using a stratified random sampling approach. This sampling method has augmented the ability of the study to generalize its outcomes to the population of older adults residing in Bushehr, Iran.

On the other hand, the present study is characterized by a cross-sectional design, which precludes the establishment of causal relationships between variables. Specifically, the inability to measure changes in the exposure and outcome variables over time limits the capacity to discern the direction of causality or determine any temporal relationship between them. Furthermore, the lack of complete dietary intake records for study participants presents a potential limitation, as it hinders the identification of nutritional confounders that may influence the outcomes of interest.

## Conclusion

To summarize, hypertension is a significant health concern closely linked to body composition and fat mass distribution throughout the body. According to this study, older adults with a higher body fat mass are more likely to develop hypertension. The research indicates that the likelihood of increased blood pressure is not only related to overall body fat but also to the accumulation of fat in specific regions such as the trunk, limbs, and android area. Consequently, when managing blood pressure in older adults, it is necessary to consider regional body fat in addition to total body fat. Hence utilizing an accurate indicator can be more beneficial in addressing the risk of hypertension, as the gynoid-to-android FM ratio was associated with a higher risk of hypertension while the relationship between the trunk-to-limb FM was insignificant. Furthermore, the importance of the location of fat accumulation should be highlighted in health care screenings and in developing health policies.

## Data Availability

The datasets used and/or analyzed during the current study are available from the corresponding author upon reasonable request.

## Abbreviations

BEH: Bushehr elderly health
AHA: American Heart Association
MMS: Measurements of Metabolic Syndrome
SBP: Systolic blood pressure
DBP: Diastolic blood pressure
BMI: Body mass index
MAP: Mean arterial pressure
LVH: Left ventricular hypertrophy
WHR: Waist to hip ratio
HTN: Hypertension
HDL: High-density lipoproteins
LDL: Low-density lipoproteins
TG: Triglycerides
TC: Total cholesterol
Hgb: Hemoglobin
RBC: Red blood cells
HbA1c: Hemoglobin A1c
NC: Neck circumferences
WC: Waist circumferences
HC: Hip circumferences
WHR: Waist to hip ratio
FM: Fat mass
FFM: Fat-free mass
LBM: Lean body mass
ASM: Appendicular skeletal muscle
FBG: Fasting blood glucose
GAT: Gluteofemoral adipose tissue
DXA: Dual-energy X-ray absorptiometry
